# Development of novel carbon-based biomedical platforms for intervention in xenotoxicant-induced Parkinson’s disease onset

**DOI:** 10.1002/bmm2.12072

**Published:** 2024-01-27

**Authors:** Jyotish Kumar, Armando Varela-Ramirez, Mahesh Narayan

**Affiliations:** 1 Department of Chemistry and Biochemistry, The University of Texas at El Paso (UTEP), El Paso, USA; 2 The Department of Biological Sciences, Border Biomedical Research Center, The Cellular Characterization and Biorepository Core Facility, The University of Texas at El Paso (UTEP), Texas, USA

**Keywords:** biomaterials, condensed matter, materials characterization, nanobiotechnology, soft matter

## Abstract

Chronic exposure to herbicides, weedicides, and pesticides is associated with the onset and progress of neurodegenerative disorders such as Parkinson’s disease (PD), Alzheimer’s disease (AD), and Amyotrophic Lateral Sclerosis (ALS). Here, we have investigated whether quinic- and chlorogenic-acid-derived Carbon Quantum Dots (QACQDs and ChACQDs, respectively) protect against a (pesticide) paraquat-insult model of PD. Our results indicated that both types of CQDs intervened in the soluble-to-toxic transformation of the amyloid-forming model protein Hen Egg White Lysozyme (HEWL). Furthermore, QACQDs and ChACQDs demonstrated antioxidant activity while remaining biocompatible in a human neuroblastoma-derived cell line (SH-SY5Y) up to 5 mg/ml and protected the cell line from the environmental neurotoxicant (paraquat). Importantly, both CQDs were found to protect dopaminergic neuronal ablation in a paraquat model of Parkinson’s disease using the nematode *C*. *elegans*. Our results are significant because both plant-derived organic acids cross the blood–brain barrier, making them attractive for developing CQD architectures. Furthermore, since the synthesis of these CQDs was performed using green chemistry methods from precursor acids that cross the BBB, these engineered bionanomaterial platforms are tantalizing candidates for preventing neurodegenerative disorders associated with exposure to environmental neurotoxicants.

## 1 | INTRODUCTION

The onset and progress of neurodegenerative disorders have been associated with the societal and industrial use of pesticides, weedicides, and herbicides (here-forth, collectively termed pesticides) and other xenobiotics.^[1–8]^ Experimental, in vitro and in vivo studies indicate that exposure to pesticides, including paraquat (PQ), rotenone, Maneb, deltamethrin, and MPTP, induces the soluble-to-toxic transformation of amyloidogenic proteins, including *α*-synuclein and Hen-Egg White Lysozyme (HEWL). Furthermore, these pesticides exert xenotoxicity in vitro and in vivo, elevate the levels of reactive oxygen species (ROS) and reactive nitrogen species (RNS) in neurons and brain tissue, drive cellular dyshomeostasis, and elicit behavioral deficits.^[9–22]^ Aberrant outputs associated with pesticide exposure include upregulation in heat-shock protein expression, elevated levels of protein ubiquitination, and the aggregation of amyloidogenic proteins, including amyloid β, *α*-synuclein, and mutant Huntingtin protein.^[23–25]^ The sequelae result in neuronal injury, neuronal demise, and neurodegenerative pathology as evidenced in clinical symptoms such as memory loss and altered gait.

Over the last few years, a host of small molecules and natural products have been experimentally tested against xenobiotic models of amylogenesis and neurodegeneration. Nevertheless, to date, these candidate molecules have failed satisfactorily to resolve pathology in clinical trials.^[26–31]^ The inability to penetrate the blood–brain barrier (BBB), or even differences in preclinical versus clinical physiology, has been implicated in unsatisfactory clinical outcomes. In this context, the need for novel platforms to intervene in the campaign against neurodegeneration continues. Carbon Quantum Dots (CQDs) are a relatively novel class of Carbon Nano Materials (CNMs) that have found numerous applications in biomedicine, environmental sensing, as switches, for drug and gene delivery, and cancer therapy.^[32–51]^ CQDs and Graphene QDs (GQDs) possess a rich framework of sp^2^-hybridized carbons.^[23]^ This feature has been found useful in intervention in pesticide-associated ROS elevation, apoptosis, necrosis and in restituting the aforementioned outputs on the trajectory to neuronal injury. Both CQDs and GQDs also intervene in the soluble-to-toxic transformation of amyloid-forming proteins, including HEWL and α-synuclein.^[23,51]^ In vitro, citric acid- and gelatin-derived CQDs have been shown to prevent the formation of mature HEWL fibrils.^[23,52–54]^ In vivo, stereotaxic injection of GQDs into the mouse brain reduced the aggregated *α*-synuclein burden, mitigated mitochondrial dysfunction, and inhibited inter-neuronal transmission of *α*-synuclein pathology upon introduction of mature *α*-synuclein fibrils.^[50]^

CQDs are deemed attractive candidates because of their potential to circumvent limitations associated with previously tested small-molecule candidates. Their ability to be easily chemically tuned, coupled with their size, makes them particularly attractive for interventional applications within the neuronal domain.

Here, we test whether QACQDs and ChACQDs are able to intervene along milestones to PD. Quinic acid qualifies as a cyclitol, a cyclic polyol, and a cyclohexane carboxylic acid. It is found in the barks of the cinchona and eucalyptus trees and is likely to be responsible for the “acidity” of coffee.^[55]^ Chlorogenic acid is an ester formed by the condensation of coffee-bean-derived caffeic acid and quinic acid.^[56,57]^ It is naturally present in the leaves of the hibiscus and bamboo trees, and in the flesh of eggplants, peaches and prunes in addition to coffee beans.^[57]^ Both acids are known to cross the BBB.^[58,59]^ Therefore, they have served as carbon precursors for the CQDs in this study to i) capitalize on the existing chemical moieties that may help penetrate the BBB post recarbonization of the precursors into CQDs and ii) improve upon their ability to scavenge ROS by enhancing the sp^2^ hybridized carbon content.

Our results reveal that both CQDs can intervene across independent milestones in the trajectory to amyloidosis. Importantly, both acids and their recarbonization into QDs via the hydrothermal process qualify as sustainable and green chemistry initiatives. Therefore, these bio-nanomaterial platforms are additionally tantalizing for applications in biomedicine.

## 2 | MATERIALS AND METHODS

### 2.1 | Chemicals and reagents

Quinic and Chlorogenic acids were purchased from Sigma Aldrich USA. Guanidine hydrochloride, ammonium bicarbonate, and potassium phosphate monobasic were purchased from Fisher Scientific. HEWL was obtained from MilliporeSigma. 1,1-diphenyl-2-picryl-hydrazyl (DPPH) was purchased from MilliporeSigma USA. Methyl viologen dichloride hydrate (PQ) was obtained from Sigma-Aldrich. All experiments were performed using analytical grade chemical and Milli-Q (MQ) water.

### 2.2 | Cell lines and supplements

Human neuroblastoma-derived SH-SY5Y cells were obtained from ATCC, USA. SH-SY5Y cells were cultured in a cell incubator at 37°C under 5% CO_2_.^[7,8,52–54]^ Trypsin (0.25%), Dulbecco’s Modified Eagle Medium/Nutrient Mixture F-12 (DMEM/F-12), and fetal bovine serum (FBS) were purchased from Sigma-Aldrich. Propidium iodide (PI) and Hoechst (H) were obtained from Life Technologies. Penicillin and Streptomycin were purchased from Gibco.

### 2.3 | Synthesis of CQDs

CQDs were separately synthesized from quinic and chlorogenic acid using the previously described hydrothermal method.^[23]^ Briefly, 150 mg of each acid was dissolved in 30 ml of deionized water and homogenously mixed in an Erlenmeyer flask using a magnetic stirrer. The solution was then transferred into a 100 ml Teflon-lined autoclave and heated at 230°C for 2 h. The resulting solution was allowed to cool to room temperature before filtration (0.22 μm syringe filter) and dialysis (1 kDa cutoff) for 10 h, resulting in a translucent brown solution. The purified solution of QACQDs and ChACQDs was lyophilized and stored at room temperature for further analysis.

### 2.4 | Physicochemical characterization

The UV/vis spectra of the CQDs (200–800 nm) were obtained using a Chemglass Life Sciences SpecMate UV-vis spectrophotometer. The particle size distribution and surface charge were measured using a Malvern Zetasizer (Nano ZS; 23). Measurements were performed by dilution of 5 mg/ml stock solution of QACQDs and ChACQDs solution with 50 μL of stock solution into 2 ml of Milli-Q water. The freshly prepared CQD solutions were sonicated for 10 min, placed in polystyrene disposable cuvettes, and measurements were recorded. For Zeta potential measurements, 1 ml of solution was prepared from a stock solution with 100 μL of the 5 mg/ml stock solution diluted to 1 ml using Milli-Q water. The solution was then placed in sample cell capillary cuvette DTS1070, measurements were recorded, and data were processed using a Malvern Zetasizer (Nano ZS).

Fluorescence emission spectra were collected using a DM45 Olis spectrophotometer. Attenuated total reflection infrared (ATR-IR) spectroscopy of QDs was performed using a Thermo Scientific Nicolet iS5 spectrometer.

### 2.5 | Cell culture

SH-SY5Y cells were cultured in a water-jacketed cell incubator at 37°C under 5% carbon dioxide (CO_2_) atmosphere.^[7,8]^ Complete culture media (DMEM/F-12) was regularly supplemented with 10% heat-inactivated fetal bovine serum (FBS) and 2% antibiotic mixture (Penicillin and Streptomycin). Cells were harvested after 70% confluency with 0.25% trypsin-EDTA and sub-cultured in a fresh T-75 flask for further experiments.

### 2.6 | Radical scavenging activity by DPPH assay

For antioxidant activity measurement of CQDs, the DPPH assay was employed.^[52–54]^ DPPH concentration was monitored by measuring the ultraviolet intensity for control and treated groups as previously described.^[52–54]^ Briefly, 0.2 mg of DPPH was freshly prepared in 1 ml of absolute ethanol and stored at 4°C as a stock solution. 0.1 mg/ml of solutions from lyophilized QACQDs and ChACQDs were prepared in methanol. In separate vials, CQD solutions were prepared in ethanol ranging from 10 to 100 μg/ml while retaining the total volume at 4.5 ml in each treatment group. Thereafter, 0.5 ml of DPPH solution (0.2 mg/ml) was added in each reaction vial for a final volume of 5 ml followed by incubation in the dark at room temperature for 30 min. Upon completion of the incubation period, UV-vis measurements were recorded to quantify the antioxidant properties at each concentration. The absorbance at 517 nm was measured to calculate the free radical scavenging activity of QACQDs and ChACQDs. In this experiment, only DPPH and ascorbic acid were used as negative and positive controls, respectively.

The percentage of antioxidant activity was calculated as follows,

$$\%\text{Antioxidant}\;\text{Activity}=\frac{\text{Absorbance}\;\text{of}\;\text{DPPH}-\text{Absorbance}\;\text{of}\;\text{Sample}}{\text{Absorbance}\;\text{of}\;\text{DPPH}}$$

### 2.7 | HEWL fibrillation inhibition ThT assay

HEWL is an amyloidogenic protein commonly used for studying protein aggregation.^[23]^ In this experiment, HEWL fibrils were prepared by dissolving 3 mg/ml of native lysozyme in potassium phosphate buffer composed of 20 mM KH_2_PO_4_ and 3 M guanidine hydrochloride (GdnHCl). Next, QACQD and ChACQD solutions were prepared at varying concentrations (1–5 mg/ml) and added to each treatment vial. Vials were left for incubation in a shaker incubator (Multi-therm, Benchmark) for 4 h at 60°C at 500 rpm. In the control and some CQD-treated samples, HEWL fibril formation was clearly seen after 5 h.^[23]^ For confirmation of HEWL fibrils, 2 ml of HEWL fibril-forming solution was placed into a quartz cuvette, and 1 ml of 20 μM ThT was introduced. Fluorescence intensity was measured for a duration of 120 s (Ex. 450 nm; Em. 482 nm) using a DM45 Olis spectrofluorometer.

### 2.8 | In vitro cytotoxicity and neuroprotective efficacy of CQDs

Cells were maintained at 37°C under 5% CO_2_. Once they attained 70% confluency, they were seeded into 96-well plates at 10,000 cells/well cell density. Cells were incubated for 24 h so that they attach to the flask surface and gain morphology. Then, cells were treated with varying QACQDs and ChACQDs concentrations (0.01–5 mg/ml) and left undisturbed for 24 h. Cells were stained with Hoechst/PI (1 μg/ml) by introducing 10 μL of the Hoechst/PI mixture into each well, followed by 1 h of incubation. The cytotoxicity profile of CQDs was examined by a multiplate reader (IN Cell Analyzer 2000 Bioimager, GE Healthcare).^[23,52–54]^ The software detected live and dead cells based on differential staining used to determine the percentage of cytotoxicity against CQD dose.

In separate experiments, 10,000 cells/well were seeded in a 96-well plate and left for 12 h incubation. Then, QACQDs and ChACQDs were introduced into cells at varying concentrations (0.01–5 mg/ml) followed by 12 h incubation. After completion of preincubation time, cells were exposed to PQ (5 mM) and incubated for 24 h. Cytotoxicity measurements were performed as aforementioned.^[23]^ The data obtained were plotted, and statistical significance was displayed in graphs with *p*-values. In these experiments, untreated cells were considered as a negative control and 10 mM hydrogen peroxide-treated cells were considered as a positive control. All confocal images were obtained using an LSM-700 confocal microscope at BBRC, UTEP.

### 2.9 | Confocal immunostaining assay

Confocal immunostaining was performed to evaluate the cytoskeletal differences between PQ (only)-treated cells and cells rescued from PQ insults.^[7,8]^ ~5000 cells/well were seeded in a 96-well plate. Cells were incubated for 12 h to allow adherence to the flask bottom. Next, cells were separately incubated with 5 mg/ml of QACQDs and ChACQDs for 12 h. After incubation, cells were exposed to PQ (5 mM) and left undisturbed for 24 h. At the end of incubation, 4% formaldehyde was added to each well, followed by 20 min incubation at room temperature. Then, formaldehyde was removed, and 200 μL of Tween 20 (0.1% for 1 h) was added to each well and washed with PBS.^[60]^ The next day, cells were stained with 50 μL of tween 20 containing 5 μg/ml of DAPI in PBS solution. 0.15 μg/ml of Alexa 568-conjugated phalloidin and 0.5 μg/ml of Alexa Fluor 488-conjugated anti-alpha-tubulin followed by 1 h incubation.^[61]^ The cells were later washed with a permeabilization solution, and finally, images were captured using an LSM-700 confocal microscope.^[62]^

### 2.10 | *C*. *elegans* culture and maintenance

The BZ555 strain of *C*. *elegans* used for the in vivo experiment was purchased from *C*. *elegans* Genetics Center at the University of Minnesota, USA. The genetically modified strain possesses GFP-expressing dopaminergic neurons (Pdat:GFP). The nematodes were maintained at 20°C in Nutrient Growth Medium (NGM) plates and cultured as previously described.^[52,53]^

Worms were age-synchronized by treating them with bleach, which also eliminates gravid adults as described. The nematode eggs were collected in a 15 ml falcon tube and then treated with 1 M NaOH, 6% RICCA sodium hypochlorite solution followed by washing with M9 buffer (5 g/L NaCl, 1 mM MgSO_4_, 2 g/L KH_2_PO_4_ and 6 g/L NaHPO_4_) up to three times to ensure that all the remaining worms were of the same age.

### 2.11 | Neuroprotective efficacy of QACQDs and ChACQDs in *C*. *Elegans*

The effects of QACQDs and ChACQDs on PQ-dependent dopaminergic neurodegeneration were assessed using the aforementioned GFP-tagged BZ555 strain of *C*. *elegans*, with a few minor adjustments to the previously reported methods.^[52,53]^ The nematodes were cultured in a 6-well plate with a 200 μL reaction volume and about 200 nematodes. The QACQDs and ChACQDs were diluted in a K-medium (5 M NaCl, 1 M KCl, 1 M CaCl_2_, 1 M CaCl_2_, and 1 M MgSO_4_ dissolved in Milli-Q H_2_O) and pre-incubated with *E*. *coli* OP50 for 24 h in a low-speed orbital shaker at 10 revolutions per minute (rpm). The nematodes were then separately incubated with QACQDs and ChACQDs solutions (initially pre-incubated with *E*. *coli* OP50) at varying concentrations at room temperature for 24 h. Milli-Q H_2_O was used to dissolve PQ (10 mM). The nematodes were then treated with 300 μL PQ (10 mM), and confocal microscopic measurements were performed at intervals of 0 h, 24 h, 48 h, and 72 h.

Following treatments, nematodes were rinsed with M9 buffer and placed on a glass slide agarose pad that contained 2% agarose in Milli-Q H_2_O and a 100 mM sodium azide solution.^[52,53]^ A Zeiss ZSM-700 fluorescence microscope was used to capture images of the nematodes. Images were obtained using a 20 × objective, and fluorescence intensity was determined using the same microscope. For each exposure group, eight worms were observed under the microscope. After PQ treatment, a decrease in green fluorescence in dopaminergic neurons was rated as evidence of neurodegeneration. Fluorescence intensities of untreated and K-medium control, PQ-treated worms, QACQDs, and ChACQDs rescued treatment groups were collected and plotted using GraphPad Prism software.

### 2.12 | Statistical analysis

The experiments with SH-SY5Y cell lines were performed in a set of triplicates to validate the experimental variability and viability.^[7,8,23,52–54]^ The statistical significance of variances in untreated and treated groups was analyzed by two-way analysis of variance (ANOVA) followed by multiple *t*-tests. The *p*-values of the untreated and treated groups are displayed in the graphs.

## 3 | RESULTS AND DISCUSSION

### 3.1 | Physicochemical characterization of quantum dots QACQDs and ChACQDs

QACQDs and ChACQDs were synthesized using the previously described one-step hydrothermal method.^[23,52–54]^ The physicochemical characterization of both CQDs was performed using a variety of spectroscopic techniques.

**FIGURE 1:**
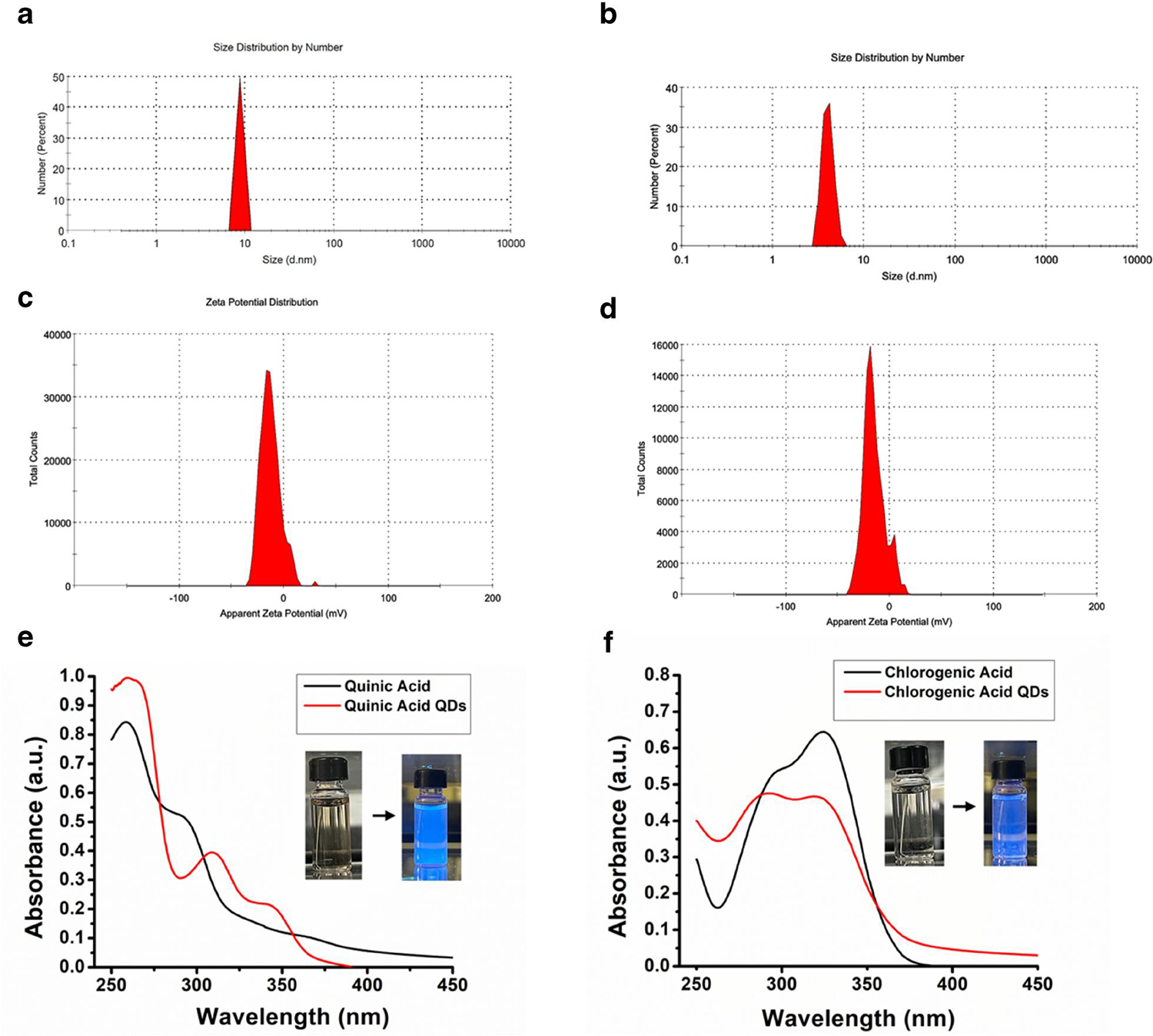
(a, b) DLS size distributions of QACQDs and ChACQDs, respectively. (c, d) represent zeta potential curves indicating a negative surface-charge distribution of QACQDs and ChACQDs, respectively. (e, f) show UV-vis absorption spectra of QACQDs and ChACQDs, respectively. Inset: Image of CQDs (e) and (f) in daylight (left) and under UV lamp (right).

The average hydrodynamic size obtained from the DLS measurements is shown in Figure 1a,b. The data reveal that QACQD and ChACQD size centered around 8.7 and 4.0 nm, respectively. The polydispersity index was found to be 0.3, which suggests that CQDs were relatively homogenous in size. The zeta potential values obtained for QACQDs and ChACQDs were −12.9 mV and −15.2 mV, respectively Figure 1c,d. The negatively charged moieties contribute to the electrostatic repulsion between the particles and prevent their aggregation, thereby contributing to CQD stability in the solvent.

The optical properties of CQDs were examined by UV-vis and fluorescence spectroscopies. Note: The CQDs appear pale yellow in white light, whereas they emit bright blue light when illuminated by UV light. Figure 1e,f demonstrate the presence of a strong absorption peak of ultraviolet light with prominences up to ~357 nm in both cases. Each absorption peak can be attributed to electronic transitions indicative of the presence of various functional groups.^[23]^ The absorption peak between 250 and 300 nm corresponds to the π→π* electronic transition of the C=C bond and that between 350 and 450 nm corresponds to the *n* →π* electronic transition of the C=C bond.

**FIGURE 2:**
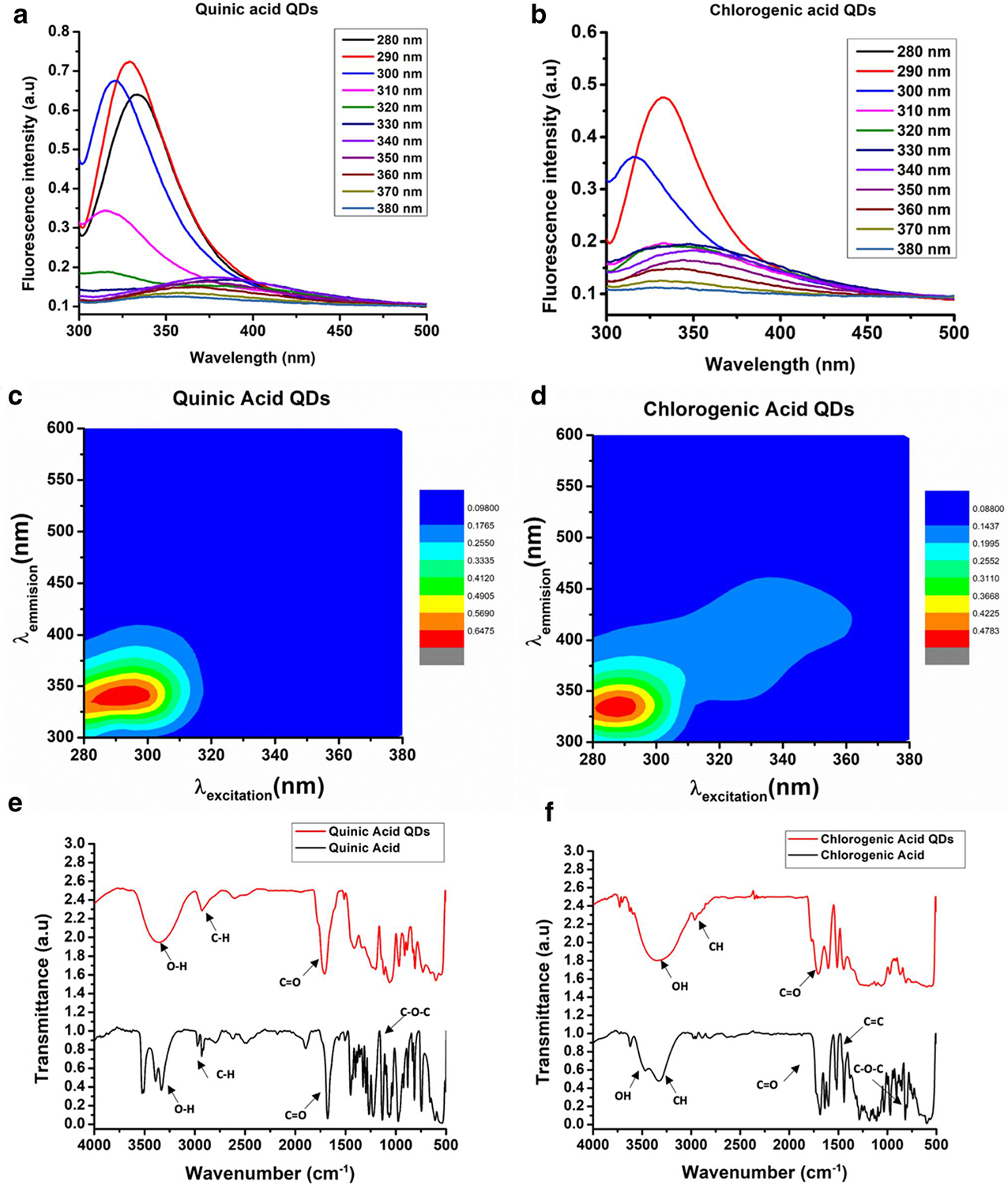
(a, b) show excitation-dependent emission spectra of QACQDs and ChACQDs, respectively. (c, d) represent the corresponding contour plots highlighting fluorescent centers in CQDs and relative quantum yields for QACQDs and ChACQDs, respectively. Panels (e, f) shows ATR-IR spectra with annotated functional groups for organic acid precursors and their corresponding CQDs.

The excitation-dependent emission fluorescence spectra of CQDs indicated a red shift for both CQDs when the excitation wavelength was varied between 280 and 380 nm. The obtained fluorescence spectra suggest the existence of multiple chemical centers with definite excitation maxima. However, and interestingly, there was a sharp reduction in the fluorescence emission intensity of QACQDs at excitation wavelengths longer than 300 nm (Figure 2a). Similarly, ChACQDs demonstrated highly reduced emission yields at excitation wavelengths of 280 nm and >300 nm. These data suggest that the absorption prominences evident from longer wavelength UV-Vis transitions have poor quantum yields.

The contour plot (Figure 2c,d) from excitation-dependent emission shows an intense emission peak when excited at 290 nm for both CQDs. While this confirms the presence of sp^2^-hybridized carbon centers even post recarbonization of the organic acids,^[23]^ the sharp decline in emission intensity at higher excitation wavelengths is reconfirmed from the very small footprint exercised by the contour.

ATR-IR spectra were obtained to underscore chemical changes in the starting materials upon recarbonization (Figure 2e,f). The spectra are indicative of the presence of functional groups, including (C-OH), (C-H), (C=C), and (C=O) in both the precursor acids and their corresponding CQDs.^[23]^ In addition, there is a broadening of the resonances in the CQDs, relative to their precursors, which indicates the presence of overlapping surface-functional water-soluble groups such as C-OH and C=O.

### 3.2 | Antioxidant properties of QACQDs and ChACQDs

**FIGURE 3:**
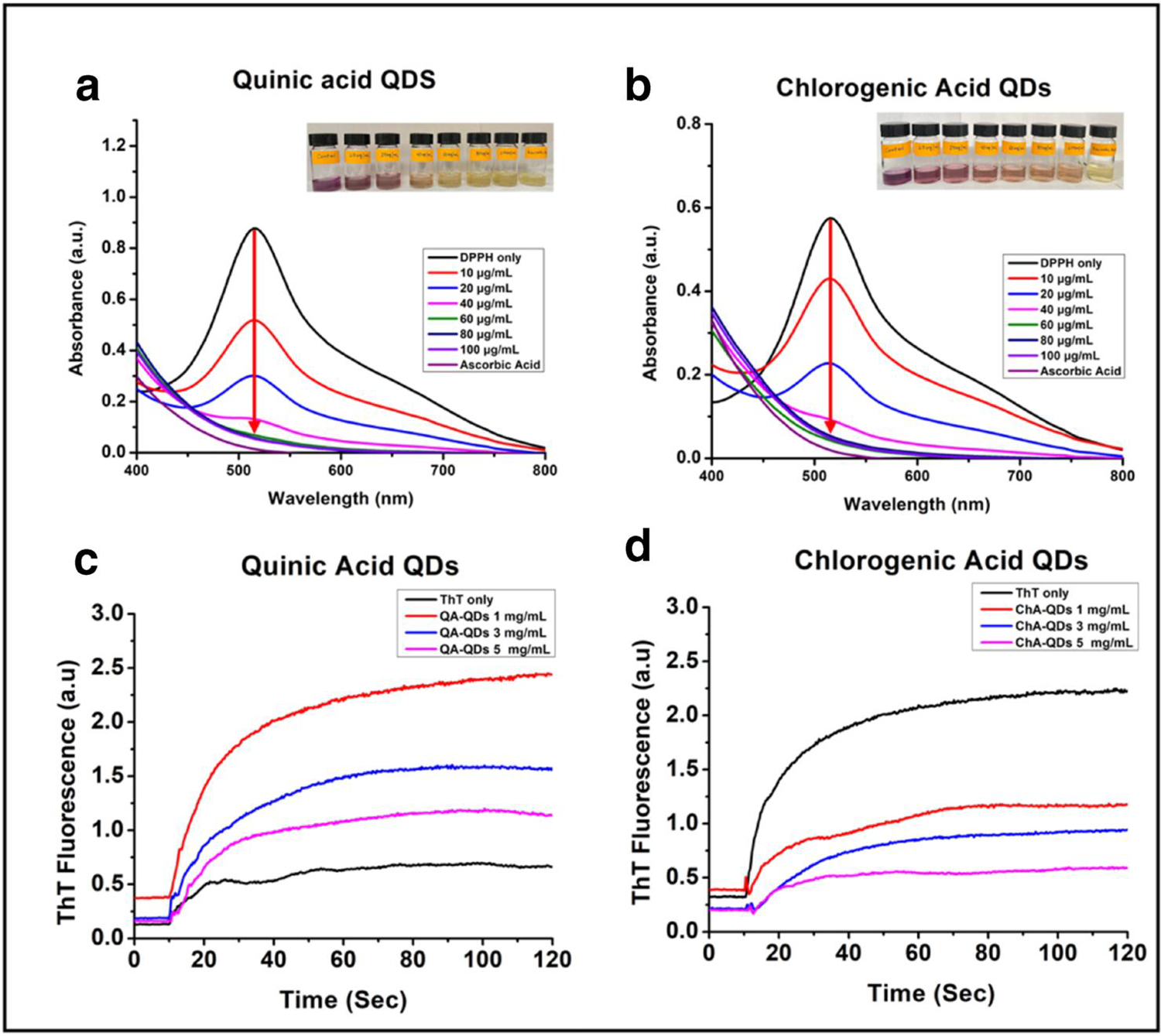
(a, b) UV-vis absorption of DPPH solution treated with QACQDs and ChACQDs (10–100 μg/ml) or DPPH alone, respectively. (c, d) ThT fluorescence of HEWL monomers pre-incubated with QACQDs and ChACQDs (1–5 mg/ml).

The antioxidant activity of QACQDs and ChACQDs was evaluated using the DPPH assay (Figure 3a,b). The assay evaluates the ability of an antioxidant to neutralize DPPH.^[52–54]^ In the presence of an antioxidant, discoloration of DPPH occurs from its oxidized form (violet) to its reduced form (yellow). The color changes from violet to yellow depending on the concentration of antioxidants. In this experiment, a CQD dose-dependent quenching of free radicals was observed when compared to the control (DPPH alone). Nearly 100% radical scavenging activity was exhibited by both QACQDs and ChACQDs at a concentration of 100 μg/ml (Figure 3a,b and insets).

### 3.3 | Prevention of soluble-to-toxic transformation of HEWL by CQDs

In this assay, HEWL was used as a model fibril-forming protein.^[23]^ ThT assay was performed to examine the impact of QACQDs and ChACQDs on the soluble-to-toxic transformation of the amyloid-forming protein. The presence of mature HEWL fibrils was detected by an increase in fluorescence upon ThT addition to the fibril solution (Figure 3c,d). The increase in ThT fluorescence intensity is characteristic of its binding to the cross-beta structure of HEWL fibrils.^[23]^ The introduction of ThT into fibrils pre-incubated with QACQDs and ChACQDs resulted in a significant diminution in ThT fluorescence intensity. Furthermore, the reduction in ThT fluorescence intensity was found to be dose-dependent (across QACQDs and ChACQDs concentrations ranging from 1 to 5 mg/ml). Even at a concentration of 1 mg/ml CQDs there was an appreciable decrease in ThT fluorescence, which indicates the potency of the CQDs in inhibiting the formation of mature HEWL fibrils. At a concentration of 5 mg/ml, both CQDs nearly eliminated the conversion of soluble monomeric HEWL to fibrils. These results indicate that both CQDs are not only capable of preventing oxidative stress associated with the onset of neurodegenerative disorders but also directly intervene in amyloid formation.

### 3.4 | In vitro cell line-based assays

**FIGURE 4:**
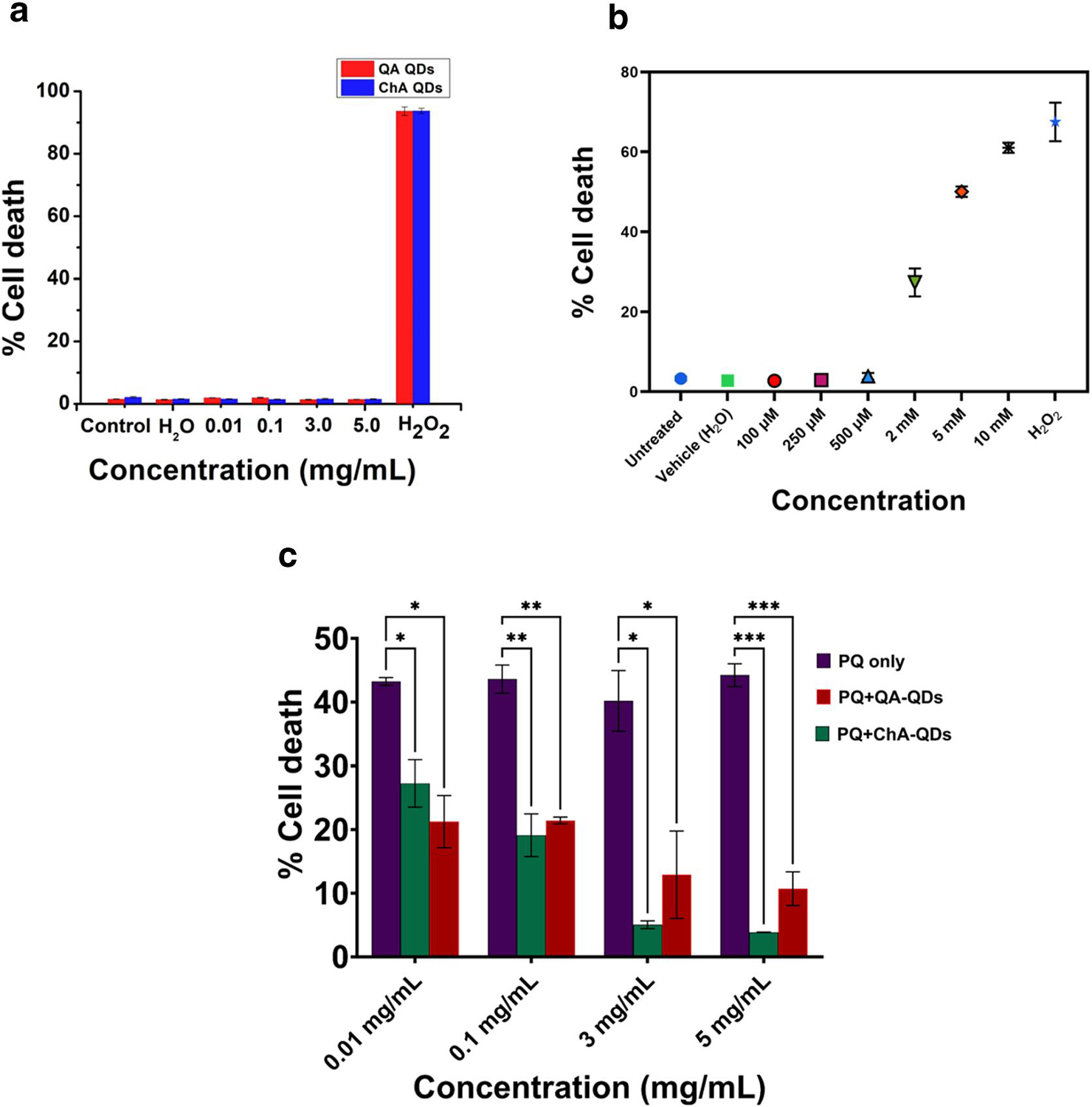
(a) Cytotoxicity profile of QACQDs and ChACQDs in SH-SY5Y cells. (b) Concentration-dependent PQ insult (100 μM–10 mM). (c) Cell rescue by pre-incubated QACQDs and ChACQDs from 5 mM PQ insult. Graphical data in (c) indicate the averages of three replicates of experiments with standard deviation values (±SD) shown in each bar. Statistical analysis was performed using Two-way analysis of variance (ANOVA) followed by multiple unpaired *t*-tests (the asterisk represents *p*-values, where *, ** and *** value indicates of *p* ≤ 0.05, *p* ≤ 0.01 and *p* ≤ 0.001 respectively).

We examined the cytotoxicity profile of QACQDs and ChACQDs in a human neuroblastoma-derived cell line (SHSY-5Y). The experiment was designed to detect live versus dead cells using Hoechst (a cell-permeable blue dye) and Propidium Iodide (membrane-compromised cell-permeable dye), respectively.^[7,8]^ Figure 4a shows the results obtained from the cytotoxicity assay. The introduction of CQDs up to 5 mg/ml did not significantly impact cell death compared to untreated and vehicle controls. Furthermore, any cell death induced by CQD treatment of cells was just a fraction of the positive control (10 mM H_2_O_2_).

The brain remains the most complex among all organs and consumes ~20% of the energy required by the body. The high energetic demands necessitate the consumption of large quantities of oxygen and nutrients including glucose into the neuronal bed, which, along with the pro-oxidant mitochondrial ETC constituent ubiquinone, make the brain particularly vulnerable to a host of pathologies, many of which are associated with mitochondrial dysfunction, neuronal inflammation and imbalances in neurotransmitters. Elevated levels of ROS are a hallmark of neurodegenerative disorders because they alter the cellular response, resulting in pathophysiological conditions such as neurodegeneration. Therefore, we tested the potency of QACQDs and ChACQDs to rescue the SH-SY5Y cell line from paraquat-induced oxidative stress. It is well known that exposure to the neurotoxin PQ initiates oxidative stress, leading to neuronal injury and subsequent demise of dopaminergic neurons.^[3,4,7,8,21,52]^ To test whether QACQDs and ChACQDs were able to intervene in a PQ-induced neurotoxicity model of PD, SHSY-5Y cells were exposed to different concentrations of paraquat (100 μM to 10 mM). A PQ dose-dependent increase in cytotoxicity was observed in comparison to untreated control, which is in agreement with previous literature (Figure 4b).^[25]^ At concentrations >500 μM, PQ was found to be toxic to cell lines, with 5 mM PQ eliciting nearly 50% of cell death.

A 5 mM PQ insult was selected to determine whether QACQDs and ChACQDs can prevent PQ-associated cell death. The results (Figure 4c) demonstrate that cells pre-incubated with QACQDs and ChACQDs (0.01–5 mg/ml) prior to PQ exposure were resistant to PQ-induced cytotoxicity as evident from the significant attenuation in cell death in comparison to the control. Additionally, the differences between PQ control and CQDs treated groups were found to be statistically significant. At a lower concentration of 0.1 mg/ml, inhibition of cell death was found to be ~15% and ~25% for QACQDs and ChACQDs, respectively. When cells were pre-incubated with a higher concentration of 5 mg/ml, the cell death rate was reduced to ~30–35% in both QACQDs and ChACQDs treated groups.

The experimental data obtained clearly reflect the prophylactic property of the two organic-acid-derived CQDs in preventing oxidative stress induced by neurotoxins such as PQ.

**FIGURE 5:**
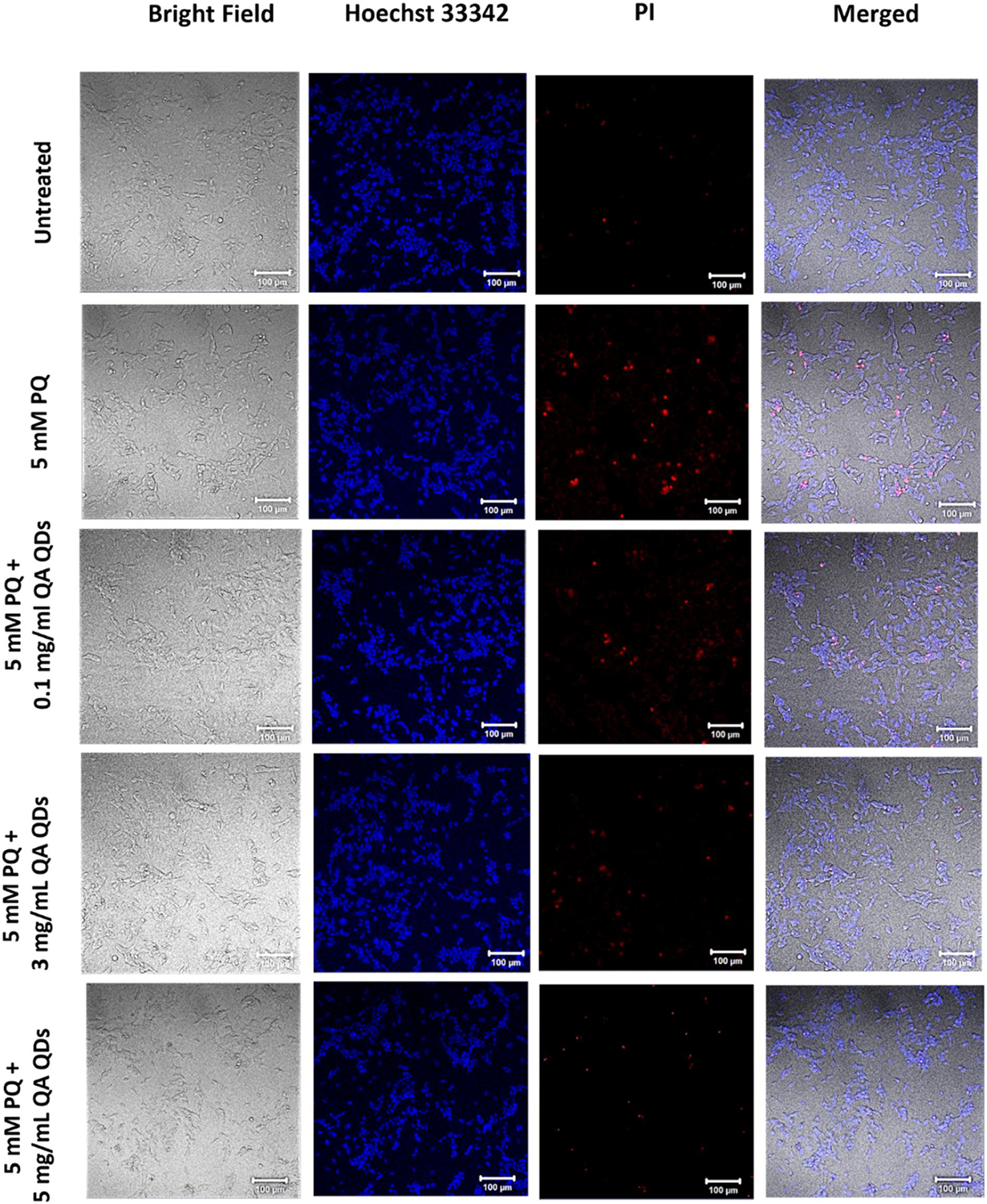
Confocal imaging of SH-SY5Y cells using Hoechst/PI showing live and dead cells in the negative control (Untreated), Positive control (5 mM PQ only), and cells pre-incubated with QACQDs (0.1 mg/ml, 3 mg/ml and 5 mg/ml) followed by 5 mM PQ.

**FIGURE 6:**
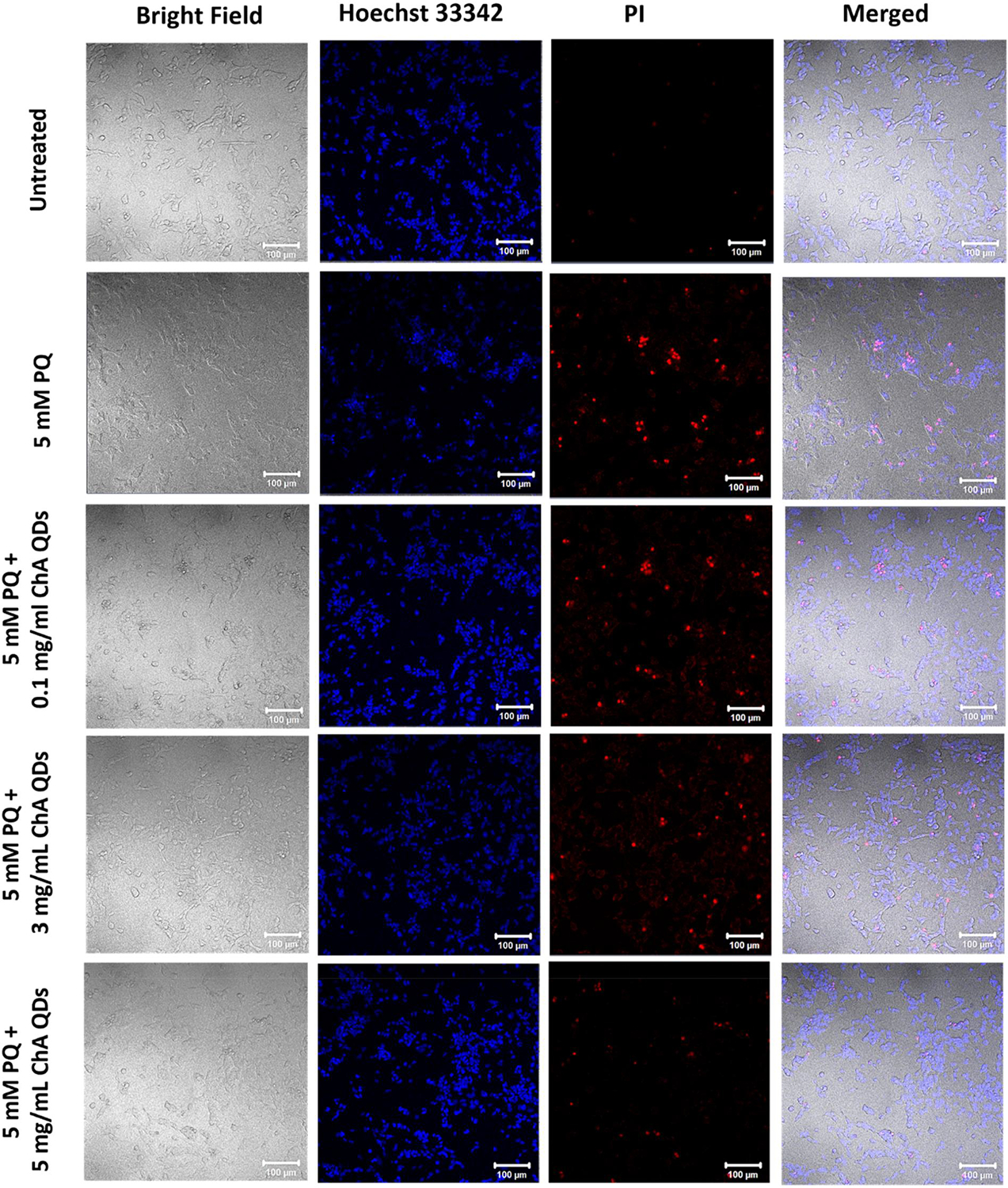
Confocal imaging of SH-SY5Y cells using Hoechst/PI showing live and dead cells in negative control (untreated), positive control 5 mM (PQ only), and cells pre-incubated with ChACQDs (0.1 mg/ml, 3 mg/ml and 5 mg/ml) followed by 5 mM PQ.

Cell rescue by QACQDs and ChACQDs was validated by confocal microscopy of SH-SH5Y cells. Live versus dead cells were identified using Hoechst (a cell-permeable blue dye) and Propidium Iodide (membrane-compromised cell-permeable dye).^[7,8]^ Cells were treated with CQDs ranging in concentration between 1 and 5 mg/ml followed by PQ treatment (5 mM). Figures 5 and 6 show a concentration-dependent increase in the frequency of live cells when cells were pre-incubated with QACQDs and ChACQDs, respectively. By contrast, as anticipated, only a few dead cells were observed in the untreated groups (negative control), and almost only dead cells were seen in wells treated with H_2_O_2_ (10 mM) as positive control.

**FIGURE 7:**
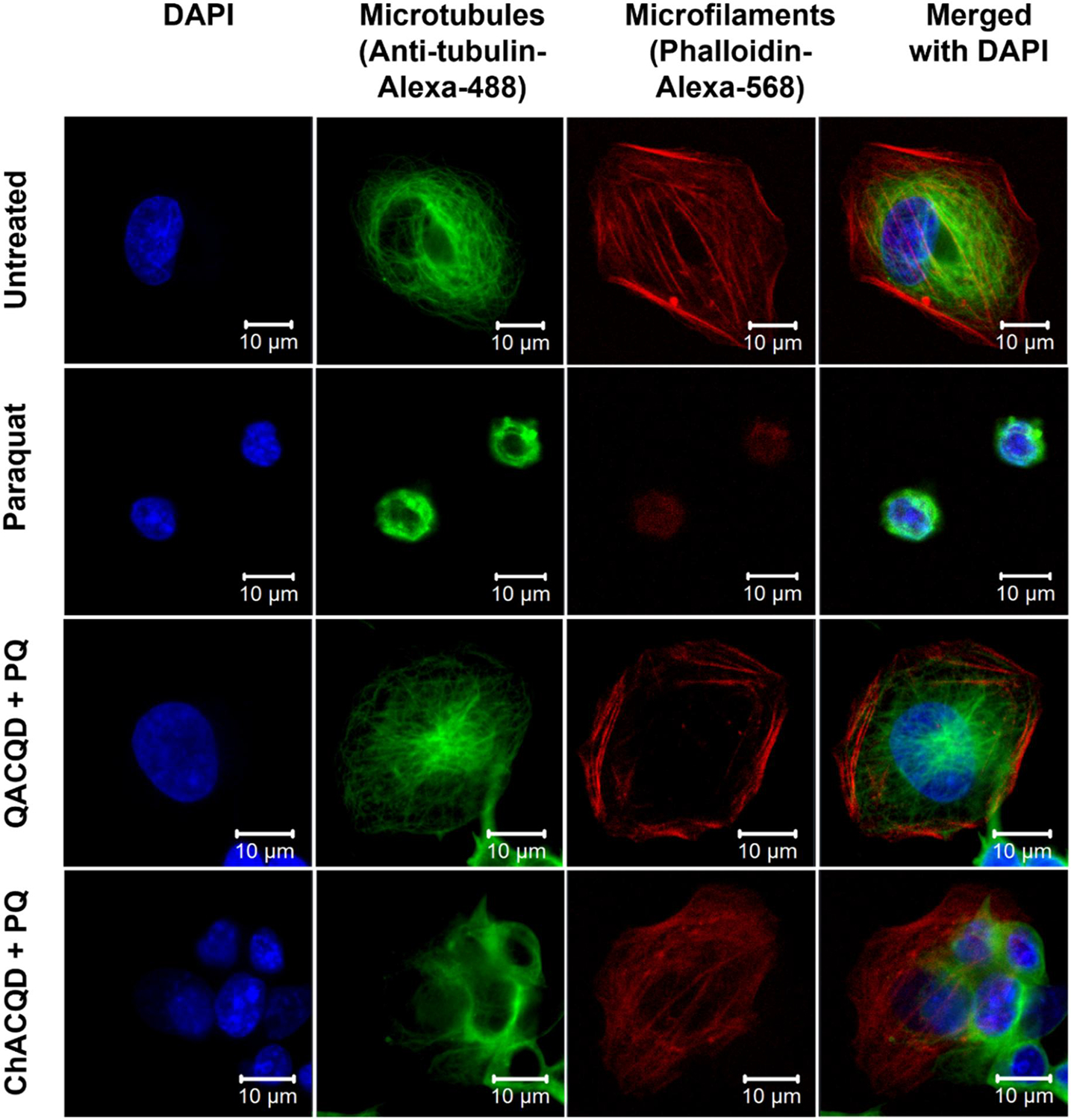
Confocal immunostaining assay for PQ (5 mM) insulted cell rescue by QACQDs (5 mg/ml) and ChACQDs (5 mg/ml). SHSY-5Y cells were stained with the nuclear stain DAPI, microtubules with Alexa Fluor 488-conjugated anti-alpha-tubulin, and microfilaments with Alexa 568-conjugated phalloidin. Merged images are depicted in the last column.

To address the cytoskeletal integrity of PQ-insulted cells and particularly cells “rescued” from PQ insult, a confocal immunostaining assay was performed.^[52–54]^ Therefore, cells were stained with nucleus- and cytoskeleton-specific staining fluorescent dyes. Nuclei were stained with fluorescent DNA intercalator DAPI, microtubules were stained with Alexa Fluor 488-conjugated anti-alpha-tubulin monoclonal antibody, and microfilaments were stained with Alexa 568-conjugated phalloidin. Figure 7 shows confocal images for untreated cells, cells pre-incubated with QACQDs (5 mg/ml) and ChACQDs (5 mg/ml) followed by PQ (5 mM) and positive control (cells treated with 5 mM of PQ only). It is evident that untreated cells exhibit extended and well-organized cytoskeleton structures with distinct microfilaments and microtubules. In contrast, PQ-treated cells lose their morphology and transform into a spherical shape with contracted microtubules and microfilaments. When PQ-insulted cells were pre-incubated with QACQDs and ChACQDs, the cells were observed to have a better morphology with distinct nuclei and organized cytoskeletal structures. The results obtained from the confocal immunostaining assay strongly support the evidence that CQDs were able to rescue the cells with PQ-induced oxidative stress.

### 3.5 | In vivo neuroprotection assay

We assessed the neuroprotective ability of QACQDs and ChACQDs in an organismal model. Nematodes (Pdat-1: GFP. BZ555 strain) were exposed to PQ, and their toxicity was quantitatively assessed using the fluorescence intensity of GFP-tagged dopaminergic neurons as previously described.^[52,53]^

**FIGURE 8:**
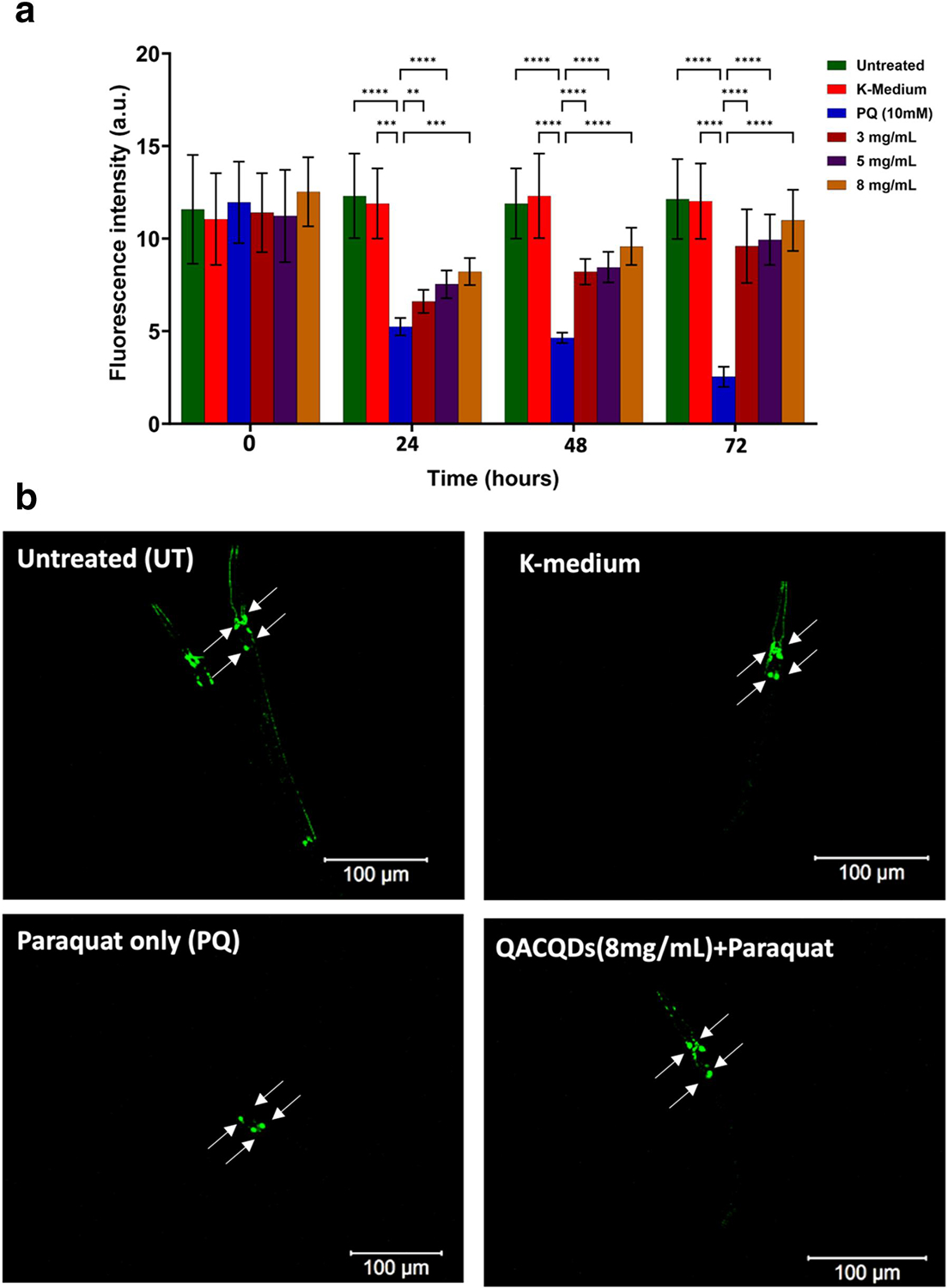
(a) Quantitative comparison of fluorescence intensity of worms exposed to QACQDs (3 mg/ml, 5 mg/ml, and 8 mg/ml) followed by PQ exposure (10 mM). Untreated and K-medium over a period of 72 h. (b) Representative images of Untreated and K-medium, PQ (10 mM), pre-incubated QACQDs (8 mg/ml) followed by PQ treatment (10 mM). Statistical analysis was performed using two-way analysis of variance (ANOVA) followed by multiple unpaired *t*-tests. The asterisk represents *p*-values, where *, **, *** and **** value indicate *p* ≤ 0.05, *p* ≤ 0.01, *p* ≤ 0.001, and *p* ≤ 0.0001 respectively.

**FIGURE 9:**
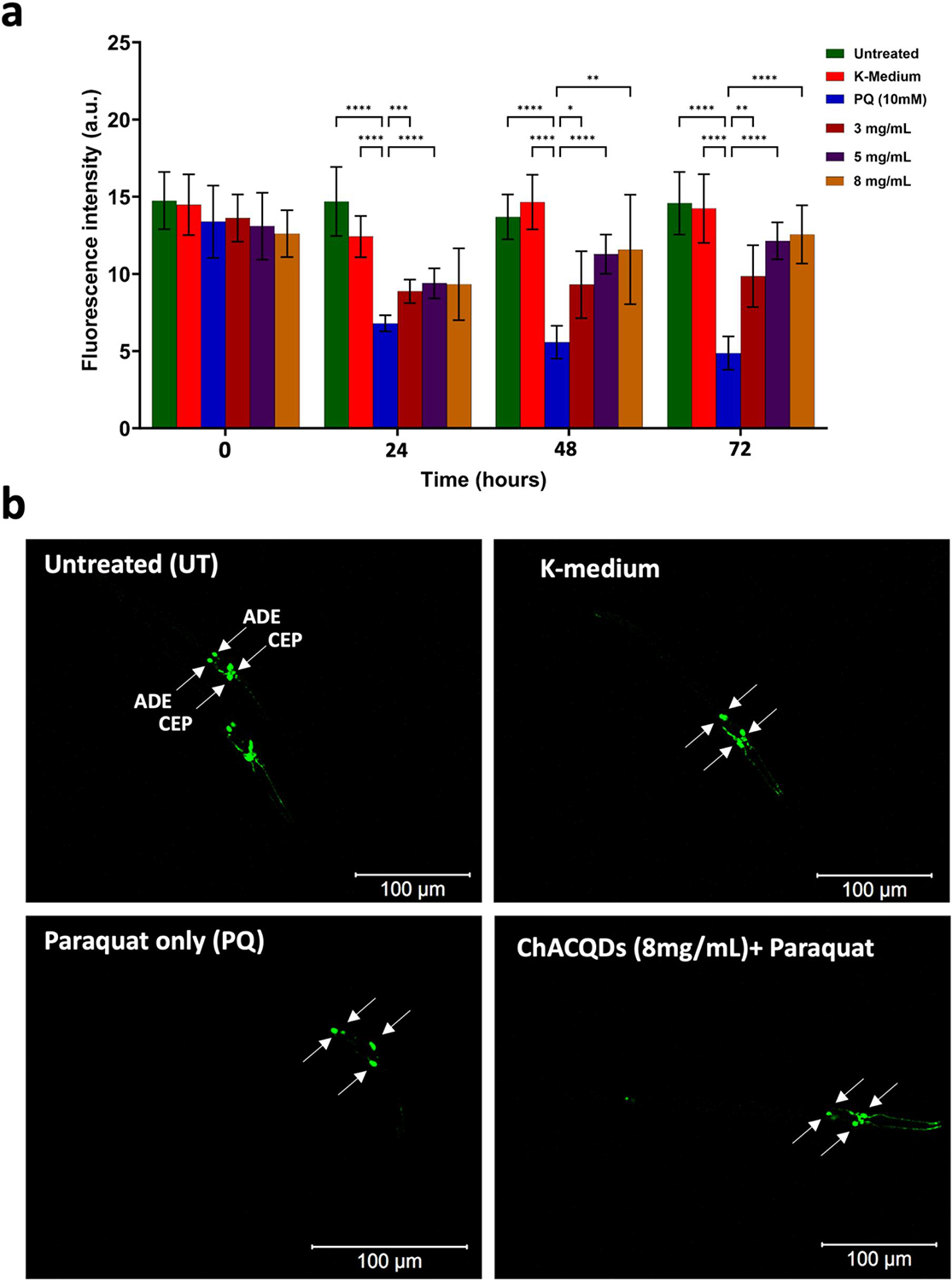
(a) Quantitative comparison of fluorescence intensity of worms exposed to ChACQDs (3 mg/ml, 5 mg/ml and 8 mg/ml) followed by PQ exposure (10 mM). Untreated and K-medium over a period of 72 h. (b) Representative images of Untreated and K-medium, PQ (10 mM), pre-incubated ChACQDs (8 mg/ml) followed by PQ treatment (10 mM). Statistical analysis was performed using two-way analysis of variance (ANOVA) followed by multiple unpaired *t*-tests. The asterisk represents *p*-values, where *, **, *** and **** value indicate *p* ≤ 0.05, *p* ≤ 0.01, *p* ≤ 0.001, and *p* ≤ 0.0001 respectively.

The results from the neuronal ablation assay are shown in Figures 8a and 9a. The data clearly reveal that the introduction of PQ (10 mM) treatment leads to a reduction in GFP fluorescence intensity (compared to the untreated and K-medium control group) in accordance with previous studies.^[52,53]^ Importantly, the administration of QACQDs and ChACQDs (3–8 mg/ml) resulted in the protection of dopamine (DA) neurons against PQ-induced neurotoxicity.

These data studies suggest that *C*. *elegans* dopaminergic neurons exhibit a significant decrease in the level of GFP expression upon exposure to PQ. By contrast, nematodes that were previously pretreated with QACQDs and ChACQDs for 24 h protected the organism from oxidative damage and neuronal ablation (by PQ).

Furthermore, all tested concentrations of CQDs demonstrated statistically significant neuroprotective effects (*p*-value of less than 0.05). That is, CQDs at concentrations of 3, 5 and 8 mg/ml exhibited a statistically significant (*p* < 0.05) neuroprotection against the loss of the GFP signal caused by PQ neurotoxicity.

Qualitative confocal microscopy images are shown in Figures 8b and 9b. The images clearly suggest that the introduction of PQ (10 mM) treatment leads to a reduction in GFP fluorescence in comparison to the untreated and K-medium control group.

## 4 | CONCLUSION

Collectively, we demonstrate the development and testing of biofunctionally active CQDs derived from sustainable sources using green chemical techniques (hydrothermal synthesis). Both QACQDs and ChACQDs demonstrate the ability to scavenge free radicals and restitute oxidative stress levels experimentally and in vitro. They are also able to mitigate the soluble-to-toxic trajectory of amyloid-forming proteins. Both features are independent of one another, and this is important because both elevated ROS and fibril formation are milestones along the trajectory to amyloidogenic disorders.

In vivo, both CQDs protect against PQ-induced ablation of dopaminergic neurons in nematodes in a dose-dependent manner. The data from this study will also form the basis for testing CQDs in vertebrate models of toxicant-induced neuronal damage. Such a study will involve crossing the BBB and adding an important and critical layer of complexity to the problem. This may necessitate further chemical tuning of the CQDs. Nevertheless, the *C*. *elegans* model has provided the necessary data for advancing these CQDs to the vertebrate model, where their ability to cross the BBB and neuroprotect rodent neurons will be determined. Simultaneously, in future studies, we will pursue an understanding of the molecular details by which both CQDs impact the soluble-to-toxic conversion of other amyloid proteins such as *α*-synuclein, amyloid β, and mHTT constructs. Is the mechanism of action dependent on the amino acid sequence of the amyloid, or are there common features across amyloids that CQDs target? Again, SAR analyses will be employed to drive such an understanding.

In conclusion, we show here that the multifactorial nature of the carbon-based frameworks is particularly tantalizing, given their independent and dual interventional roles in neurodegenerative onset and pathogenesis. The findings herein position both functional materials for testing in vertebrate models of neurodegenerative disease.

## Data Availability

Data availability statement not applicable.
